# The Impact of Sources of Variability on Parametric Response Mapping of Lung CT Scans

**DOI:** 10.18383/j.tom.2015.00148

**Published:** 2015-09

**Authors:** Jennifer L. Boes, Maria Bule, Benjamin A. Hoff, Ryan Chamberlain, David A. Lynch, Jadranka Stojanovska, Fernando J. Martinez, Meilan K. Han, Ella A. Kazerooni, Brian D. Ross, Craig J. Galbán

**Affiliations:** Departments of 1 Radiology and; 2 Internal Medicine, University of Michigan, Center for Molecular Imaging, Ann Arbor, MI;; 3 Imbio LLC, Minneapolis, MN;; 4 National Jewish Health, Denver, CO; and; 5 Weill Cornell Medical College, New York, NY

**Keywords:** parametric response map, lung disease, quantitative CT, sources of error

## Abstract

Parametric response mapping (PRM) of inspiration and expiration computed tomography (CT) images improves the radiological phenotyping of chronic obstructive pulmonary disease (COPD). PRM classifies individual voxels of lung parenchyma as normal, emphysematous, or nonemphysematous air trapping. In this study, bias and noise characteristics of the PRM methodology to CT and clinical procedures were evaluated to determine best practices for this quantitative technique. Twenty patients of varying COPD status with paired volumetric inspiration and expiration CT scans of the lungs were identified from the baseline COPDGene cohort. The impact of CT scanner manufacturer and reconstruction kernels were evaluated as potential sources of variability in PRM measurements along with simulations to quantify the impact of inspiration/expiration lung volume levels, misregistration, and image spacing on PRM measurements. Negligible variation in PRM metrics was observed when CT scanner type and reconstruction were consistent and inspiration/expiration lung volume levels were near target volumes. CT scanner Hounsfield unit drift occurred but remained difficult to ameliorate. Increasing levels of image misregistration and CT slice spacing were found to have a minor effect on PRM measurements. PRM-derived values were found to be most sensitive to lung volume levels and mismatched reconstruction kernels. As with other quantitative imaging techniques, reliable PRM measurements are attainable when consistent clinical and CT protocols are implemented.

## Introduction

Lung densitometry by x-ray computed tomography (CT) is sensitive to the alterations in lung parenchyma as a result of the onset and progression of chronic obstructive pulmonary disease (COPD). Quantitative CT-based measures of lung disease are well characterized (1) yet have not fully transitioned into routine clinical use, where physiological assessment and clinical patient-reported parameters remain the standard of care for COPD. However, large clinical studies such as COPDGene (2) and SPIROMICS (3) have been undertaken using a standardized chest CT image acquisition protocol for disease phenotyping and for assessing progression. With longitudinal CT data collection in process, quantitative CT metrics must be fully characterized to minimize measurement variability between serial examinations.

Emphysema and air trapping are 2 components of COPD that are measured on CT images. Emphysema is measured as the percentage of lung with voxels below −950 Hounsfield units (HUs) (4, 5). Air-trapping measures, which quantify the extent of both emphysematous- and nonemphysematous-diseased tissue, include the percentage of lung with voxels below −856 HU on expiration scans (2) and the ratio of mean lung density on inspiration-to-expiration CT scans (6). In 2012, a voxel-based technique called parametric response mapping (PRM) was shown to discriminate between these 2 forms of air trapping when applied to paired and spatially registered inspiration and expiration CT scans (7). PRM applies previously defined thresholds for the emphysema index (< −950 HUs) and air trapping (< −856 HUs) on spatially aligned inspiration-to-expiration CT scans (8, 9). This allowed individual voxels within the lung parenchyma to be classified as normal (PRM^Normal^), emphysematous (PRM^Emph^), and nonemphysematous air trapping, referred to as functional small airways disease (PRM^fSAD^). Whereas PRM^Emph^ is analogous to earlier density-based emphysema indices, PRM^fSAD^ represented regions of air trapping that were nonemphysematous (7, 10). Like other quantitative CT measures, PRM may be influenced by differences among scanner types, patient compliance, scan acquisition protocols, scanner HU drift, and the selection of reconstruction kernels. Therefore, reducing CT-based metric variability by identifying, quantifying, and eliminating (when possible) the sources of variability (11, 12) is required to determine the detection limits for assessing true changes in COPD patient CT scans.

**Figure 1.**
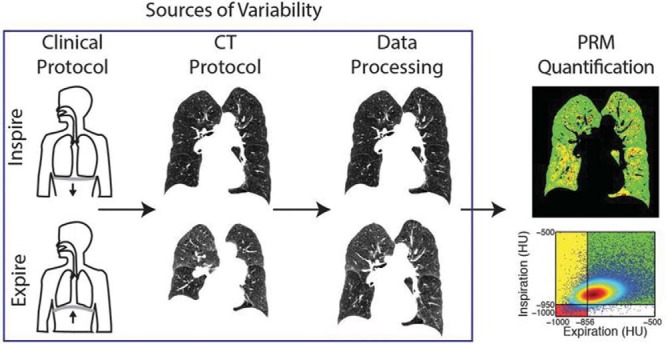
Schematic of modes of variability at various stages in the PRM workflow. Clinical protocol consists of patients being trained to hold their breath at full inspiration and full or relaxed expiration. CT protocol guides acquisition at both points using site-specific CT systems, acquisition parameters, and reconstruction kernels. Data processing consists of lungs from the serial CT scans being segmented from the thoracic cavity and then spatially aligned to a single geometric frame using site-specific algorithms. PRM quantification is performed by classifying voxels with paired HU values as normal parenchyma (green voxels), functional small airways disease (yellow voxels), or emphysema (red voxels).

This study evaluates the sensitivity of PRM to sources of variability present in a standardized CT clinical acquisition and processing protocol that may affect quantitative CT measurements (Figure 1). Although many of the parameters investigated had a minimal impact on expected PRM values, this work demonstrates the effect of lung inspiration/expiration volume at CT acquisition on PRM measurements. These results reveal that establishing the correct lung inhalation and exhalation volumes during image acquisition is the most important quality control measure for longitudinal CT imaging.

## Methods

### Study Participants

Data used in this study were obtained under an institutional review board–approved protocol, and all participants involved provided written consent as part of the COPDGene clinical trial (2). Participants were separated into groups of 4 based on Global Initiative for Chronic Obstructive Lung Disease (GOLD) (13) stages 0 to 4 (Table 1). Baseline clinical and CT data were used from a total of 20 randomly selected participants along with participant characteristics and pulmonary function test results.

**Table 1.** Subject Characteristics

Parameter	GOLD 0	GOLD 1	GOLD 2	GOLD 3	GOLD 4
Sex (female/male)	1/3	1/3	2/2	3/1	2/2
Age (y)	54.1 (12.6)	67.4 (8.5)	62.4 (11.8)	57.2 (8.0)	67.0 (11.6)
Height (cm)	173.0 (16.0)	171.0 (4.0)	170.8 (13.4)	167.8 (5.8)	170.5 (6.6)
Weight (kg)	77.4 (20.1)	76.2 (11.4)	72.7 (25.7)	80.2 (39.2)	63.8 (10.8)
BMI (kg/cm^2^)	25.4 (3.0)	26.0 (3.1)	24.3 (6.16)	28.3 (13.6)	21.8 (1.9)
TLC (from CT)	5.9 (1.1)	6.3 (1.1)	6.1 (1.31)	6.2 (0.4)	7.3 (0.3)
RV (from CT)	3.0 (0.3)	2.9 (0.3)	3.4 (.32)	4.2 (0.6)	5.6 (0.6)
Percentage of FEV1 predicted	102.3 (14.1)	89.3 (4.3)	70.0 (11.0)	37.5 (7.6)	19.6 (3.7)
Percentage of FVC predicted	99.3 (14.7)	107.5 (10.8)	101.3 (20.3)	85.8 (9.0)	48.5 (13.8)
FEV1/FVC	0.8 (0.06)	0.6 (0.07)	0.5 (0.10)	0.3 (0.04)	0.3 (0.04)

Values are mean ± SD.

Abbreviations: BMI, body mass index; FEV1, forced expiratory volume in 1 second as a percentage of predicted; FVC, forced expiratory vital capacity; RV, residual expiratory volume computed from CT; TLC, total lung capacity computed from CT.

### CT Data Acquisition

Quantitative CT data, measured in HUs, were acquired at full inspiration (total lung capacity [TLC]) and at relaxed expiration (functional residual capacity [FRC]) as defined in the COPDGene protocol (2). Scans used were obtained from different scanners involved with image acquisition in the COPDGene trial (Table 2). CT data were reconstructed using both sharp and standard kernels. All CT scans were linearly corrected using predefined mean HU values for blood (50 HUs) and air (−1000 HUs) (14, 15).

**Table 2.** CT Manufacturers and Brands

N	Manufacturer	Scanner
1	Siemens	Sensation 16 (0.75-mm voxels)
3	Siemens	Sensation 64 (0.75-mm voxels, 3 reconstructions)
6	Siemens	Definition (0.75-mm voxels)
4	GE	LightSpeed VCT (0.625-mm voxels)
6	GE	LightSpeed 16 (0.625-mm voxels)

### CT Data Analysis

The mean HU of air and aortic blood was measured from the paired CT data acquired from different vendor systems and reconstruction kernels. Volumes of interest (VOIs) were manually contoured at approximately a 10-mm radius or smaller as needed within the ambient air outside the patient, the blood within the aorta, and the air inside the trachea.

PRM was applied to paired inspiration/expiration CT scans as previously reported (7). In brief, the lung parenchyma was segmented from the thoracic cavity and airways. Inspiration and expiration image volumes were spatially aligned to a single geometric frame using a thin-plate spline with mutual information as an object function. Each parenchymal voxel (ie, the smallest unit of volume in an image data set) was classified using the emphysema index and air-trapping thresholds, defined by COPDGene as −950 HUs on the inspiration scan and −856 HUs on the expiration scan, respectively, and used for generating joint-density histograms of paired parenchymal HU values of the voxels. This allowed each voxel to be classified as normal (green), emphysematous (red), or nonemphysematous airflow obstruction (also referred to as functional small airways disease) (yellow). Relative volumes for each class were calculated by summing all voxels within a classification and normalized to the total lung volume.

### Generation of Simulated CT Data

The impact of inadequate lung ventilation during CT acquisition on PRM measurements was evaluated using simulated CT data from 5 subjects, each representing GOLD 0 through 4. This was performed by spatially deforming the expiration lung scans using a mass-preserving, diffeomorphic transform to model a series of exhalation lung volumes in 10 steps from expiration to full-inspiration volumes acquired at TLC (16). Mass was preserved by adjusting the HU values for volume changes by multiplying each voxel by the local Jacobian of the warping transform. Simulations used linear control point trajectories between ∼50 control points per lung identified on both the expiration and inspiration lungs. PRM was applied to the paired original inspiration and simulated expiration CT data as previously described.

Simulated CT data were also generated to evaluate the impact of spaced thin-slice expiration CT data on PRM measurements. Gapped CT data were generated from contiguous whole-lung CT scans by subsampling the more widely spaced thin-slice images, resulting in an axial spacing of 0.625, 1.25, 2.5, 5, and 10 mm or 0.75, 1.5, 3.0, 6.0, and 12 mm depending on the original reconstruction. PRM was applied to the paired simulated spaced expiration and original inspiration CT data as previously described.

Simulations were performed to test the influence of misregistration on PRM metrics. A known high-quality registration solution was defined by 113 feature points that served as basis points for thin-plate spline transforms. Random perturbations of this feature set were generated and used to compute transforms that created misregistered solutions. Simulated target deformations ranged from 0 to 30 mm (the approximate distance the diaphragm moves between inspiration and expiration; a mean of ∼15 mm would be no registration), where any simulations with folding were removed. Included deformations had a mean feature displacement of 0 to 6.6 mm, resulting in features that moved up to a mean of approximately 10 voxels with a 40-voxel regional deformation maximum. PRM was calculated at each registration solution, and the results were analyzed to determine how local misregistrations contributed to PRM metric variability.

### Statistical Analysis

Ambient air, tracheal air, and aortic blood HU values between vendor systems were compared using an unpaired Student *t* test. A paired Student *t* test was used to compare differences in HU values in the 3 VOIs between reconstruction kernels. Bland–Altman analysis was performed to illustrate differences in the individual PRM metrics between sharp and standard reconstructed CT data. All statistical analyses were performed using IBM SPSS Statistics version 21.

## Results

### Impact of Lung Ventilation Variability

**Figure 2.**
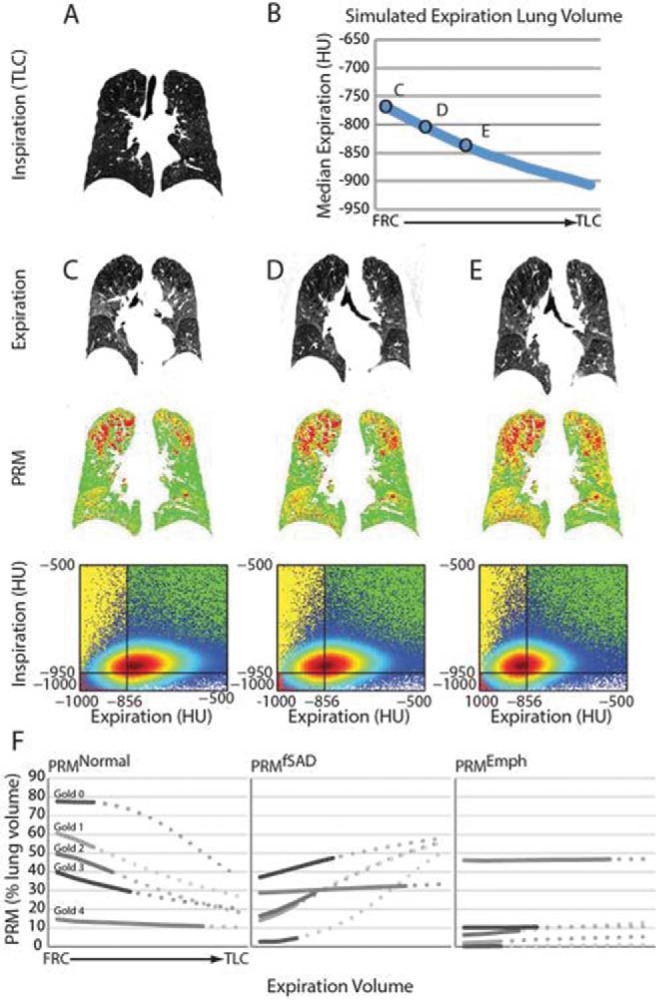
Impact from variability in lung ventilation on PRM. PRM measurements from a patient diagnosed with GOLD stage 2 COPD show the influence of variable expiration resulting from inadequate training or disease. CT data was acquired at full inspiration (A) and relaxed expiration (C) (ie, FRC). Representative CT lung slices, PRMs, and corresponding lung HU joint-density scatter plots are presented at FRC and simulated expiration volumes (C) increased by 20% (D) and 40% (E) from FRC (C). The effective change in median HU values at various expiration volumes are presented in (B) and correspond to a downward shift of the peak in the joint-density plot on the expiration axis, resulting in more tissue classified as PRM^fSAD^ and PRM^Emph^. The resulting changes in PRM^Normal^, PRM^fSAD^, and PRM^Emph^ are shown for 5 sample cases at relaxed expiration (F), where solid lines depict a 0% to 20% change in expiration volumes simulated from FRC to TLC and dotted lines represent further shifts in expiration volumes toward TLC.

Simulated expiration lung volume increase (Figure 2C–E) resulted in a drop in the mean HU density of the lung (Figure 2B). PRM analysis of the original inspiration and expiration at various simulated volumes resulted in a decrease in PRM^Normal^ and increase in PRM^fSAD^. As shown in Figure 2, this trend resulted from a shift of the joint-density histogram toward less attenuation along the expiration axis (*x*-axis). Those cases with a large dynamic range between inspiration and expiration volumes, that is, GOLD stages 0 and 1, demonstrated the most sensitivity to insufficient expiration ventilation in PRM metrics (Figure 2F). Nevertheless, realistic variability in expiration volumes from FRC is typically around 20% (Figure 2F, solid lines). Deviation in expiration volumes within this range (0%–20% of FRC) resulted in only subtle changes in PRM metrics that were mainly observed in GOLD stage 1 through 3 cases. PRM^Emph^, classified primarily by the −950-HU threshold on inspiration scans, varies only slightly with exhalation volume changes (Figure 2F). Simulations that resulted in inaccurate PRM^Emph^ measurements were observed from the anticipated effect of insufficient inspiration during a TLC maneuver (data not shown).

### Impact of Reconstruction Kernel and Scanner Manufacturer

**Figure 3.**
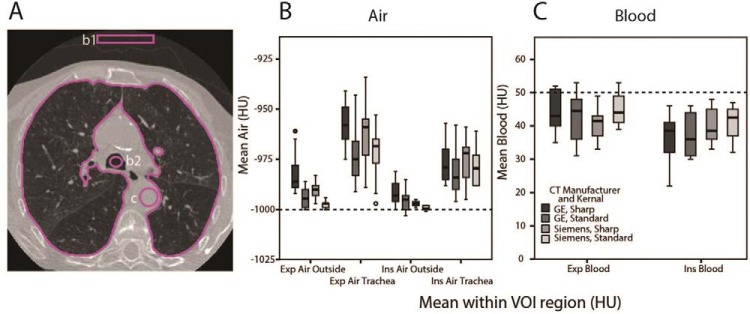
Impact of CT vendor and reconstruction kernel. HU values are measured in ambient air (b1), tracheal air (b2), and aortic blood (c) for 2 scanner brands and standard and sharp reconstructions. The 3 regions of interest are depicted in a representative axial CT slice (A). Box plots are presented for the HU values of air (B) and aortic blood (C) obtained from CT data acquired from different CT vendors and reconstruction kernels. For reference, ideal air and water attenuation values should be −1000 HUs and 0 HUs, respectively, with a target blood value of 50 HUs (27). The lines on the box plot are as follows: center line, median; bottom and top boxes, 25% and 75% quantiles, respectively; bottom and top bars, 1.5 times the box size or the minimum value if no values fall in that range. Note for normally distributed data approximately 95% of the data are expected to lie between the distal fences.

**Figure 4.**
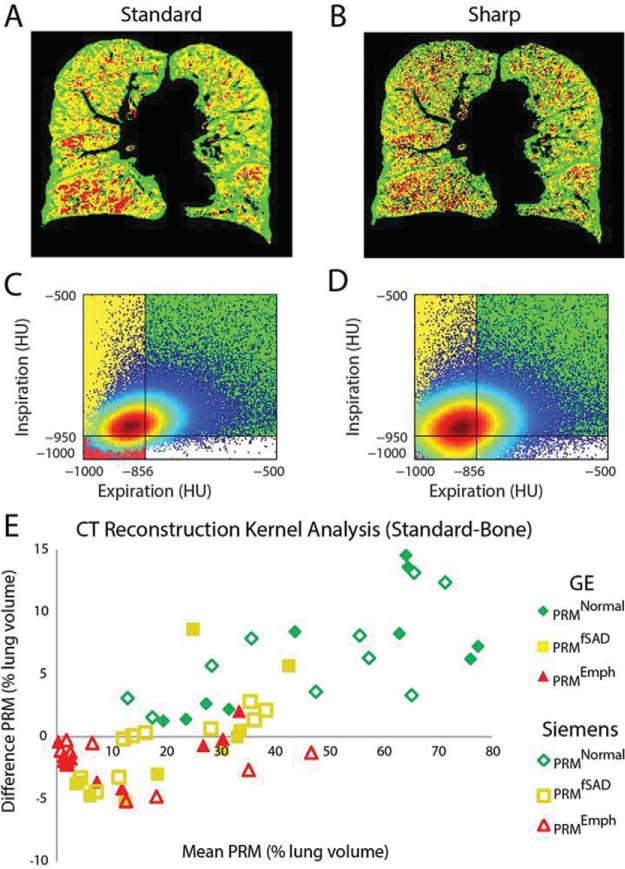
Impact of reconstruction kernels on PRM. Standard kernel reconstruction results in the smoothing of PRM (A) compared with a bone reconstruction (B). The noisier sharp reconstruction results in a broader distribution for the HU joint-density histogram (D) than the standard reconstruction (C). Bland–Altman analysis (E) shows that standard reconstructions result in an increase in PRM^Normal^ and decrease in PRM^Emph^ classifications relative to bone reconstructions regardless of scanner type. PRM^fSAD^, however, is reduced in mild COPD but increases in more severe diseases with standard compared to bone reconstructions for both scanner types.

The impact of reconstruction kernel and scanner type on HU values is demonstrated in ambient and tracheal air and aortic blood. As shown in Figure 3, mean values of ambient air differed significantly between reconstructed kernels in both inspiration (vendor 1, *P* = .001; vendor 2, *P* = .001) and expiration (vendor 1, *P* = .001; vendor 2, *P* < .0001) CT scans. In addition, expiration tracheal air mean HU values were also found to vary between reconstruction kernels (vendor 1, *P* < .0001; vendor 2, *P* = .01). Negligible differences in mean HU values were observed for aortic blood irrespective of vendor. Differences in reconstruction kernels were evident in the PRM measurements from the same cases (Figure 4). Soft-tissue reconstructions (ie, standard) resulted in a tighter cluster of lung HU voxel-paired values (Figure 4A, C), whereas sharp bone reconstructions contained more noise, resulting in a broader distribution of the voxel joint-density histogram (Figure 4B, D). Bland–Altman analysis (Figure 4E) of the data revealed that PRM metric variability derived from standard and sharp kernels. Elevated levels in PRM^Normal^ resulted in differences as high as 15% relative lung volume between reconstruction kernels, with the standard kernel generating larger PRM values than the sharp kernel as indicated by positive differences. In contrast, PRM^fSAD^ and PRM^Emph^ were found to have both positive and negative differences between reconstruction kernels. These results illustrate the complexity of the reconstruction kernel's impact on PRM measurements.

### Impact of Slice Interval

**Figure 5.**
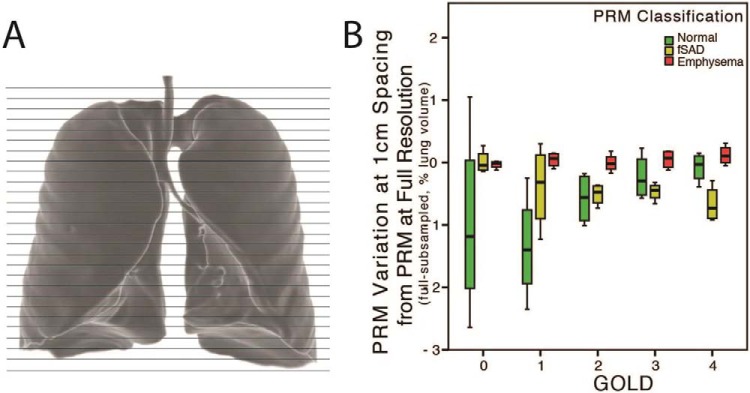
Impact of 10 mm slice intervals on PRM. Spacing between slices is illustrated in (A). PRM differences from full resolution are presented per GOLD stage for a standard reconstruction kernel (B). Variations in PRM^Normal^ are greater for mild COPD. PRM^fSAD^ varies the most in mild COPD, whereas similar but small variations in all 3 metrics are seen in moderate-to-severe COPD. The lines on the box plot are as follows: The lines on the box plot are as follows: center line, median; bottom and top boxes, 25% and 75% quantiles, respectively; bottom and top bars, 1.5 times the box size or the minimum value if no values fall in that range. Note for normally distributed data approximately 95% of the data are expected to lie between the distal fences (n = 4 per GOLD stage).

The impact of noncontiguous CT scans with a 10-mm gap spacing on PRM were evaluated next (Figure 5). Figure 5A shows a schematic of the simulated distribution of CT slices with 10-mm gaps superimposed on a full-inspiration lung scan. Overall, mean differences in PRM values between gapped and full-resolution (ie, contiguous slices) CT data were generally small, with moderate-to-severe emphysema (<1%) being the least affected. Differences were found to be slightly elevated in GOLD stage 0 (PRM^Normal^ = <3%) and GOLD stage 1 (PRM^Normal^ = <2%; PRM^fSAD^ = <1.5%) participants (Figure 5B). Nevertheless, differences in all PRM metrics were relatively low and likely resulted from the diffuse nature of COPD in the cases analyzed.

### Impact of Image Registration

**Figure 6.**
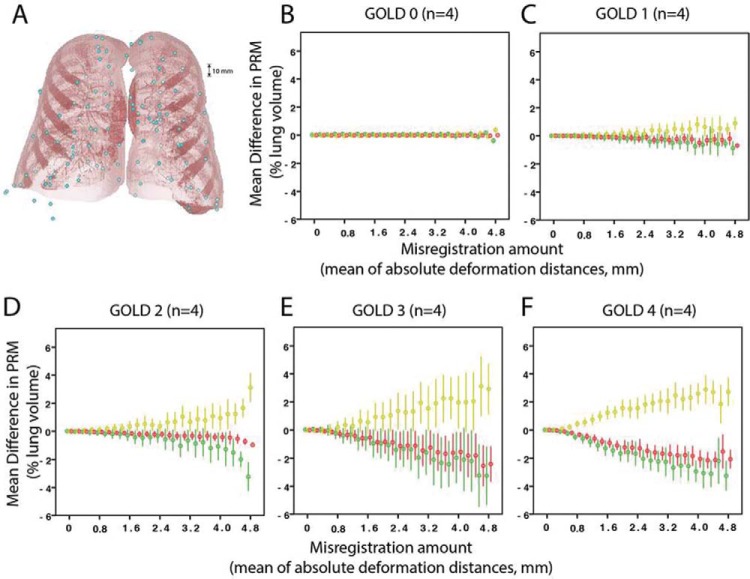
Impact of misregistration on PRM. Misregistration is simulated using a feature set (aqua dots) shown on a sample lung volume (A) that is moved slightly to deform a good registration solution (4 each; Gold stages 0 to 4). Changes in PRM increase as the mean absolute misregistration across the feature set increases. PRM changes are pooled by GOLD stage and represent mean ± SD differences (B–F). Note that mean changes of 2.5 mm may include local deformation regions of up to 7.5 mm or approximately 10 voxels.

**Figure 7.**
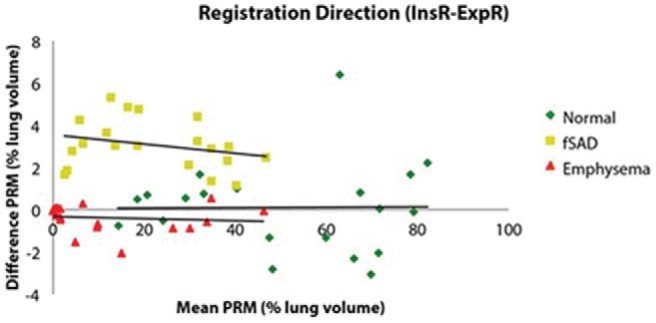
Impact of registration direction on PRM. Registering the inspiration to the expiration resulted in higher percentages of tissue identified as air trapping. Because tissue compresses upon expiration, tissue with functional small airways disease compressed less and remained a larger percentage of the total lung volume than if measured on the inspiration volume. Differences of PRM^Normal^ and PRM^Emph^ were close to 0, but PRM^Normal^ has a slightly larger 95% CI.

Simulations were performed to assess how sensitive the PRM metrics were to the misregistration of paired whole-lung CT data. Widespread local misregistration showed progressive variability of PRM metrics as a function of increasing deformations (Figure 6). The largest differences in PRM were observed in GOLD stage 3 and 4 participants, where all 3 classifications provide significant contributions to the lung volume. Overall, mean misregistrations of 2.5 mm (maximum deformation of 7.5 mm) yielded PRM differences of up to 1% of the lung. Furthermore, we also investigated and quantified the impact of registration direction on PRM because this can affect overall PRM results. Registering the inspiration to the expiration images resulted in higher percentages of tissue identified as air trapping (Figure 7). Because tissue compresses upon expiration, tissue with functional small airways disease compressed less and remained a larger percentage of the total lung volume (mean: PRM^fSAD^ = 3.1; 95% CI: 0.8, 5.4) than if measured on the inspiration volume. Differences of PRM^Normal^ and PRM^Emph^ were close to 0, but PRM^Normal^ had a slightly larger 95% CI (mean: PRM^Normal^ = 0.1; 95% CI: −4.1, 4.2) (mean: PRM^Emph^ = −0.4; 95% CI: −1.6, 0.8).

## Discussion

The consistency of PRM measurements for longitudinal application was evaluated for several prevalent sources of variability in the PRM-processing pipeline. Most of these steps introduced negligible variability in PRM. Nevertheless, significant effects were found as a result of inadequate lung ventilation during image acquisition. The clinical protocol for CT acquisition introduces various sources for HU variability that include proper lung ventilation and thin-sliced gapped CT acquisitions during expiration. The more significant of these 2 effects was the volume of lungs at image acquisition (TLC, FRC). PRM, with its use of inspiration and expiration CT scans, is susceptible to both volumes. A recent study has found that at lung volumes above 90% of vital capacity, emphysema Perc1 measures (first percentile of inspiratory attenuation distribution) varied negligibly (17). These results are in agreement with our PRM^Emph^ values generated from simulations with slightly varying inspiration lung volumes from TLC (data not shown). Moreover, previous studies have delineated the interscan measurement variability caused by inspiration differences among scans (18) along with a proposal for using optimal protocols for CT surveillance of emphysema in a lung cancer screening environment (19). However, for expiration lung volumes, small corrections have been applied to low-attenuation analysis with a threshold of −856 HUs on the expiratory image (LAA_−856E_) by adjusting the thresholds based on the degree of deviation from the normal population in the difference of 90th percentile attenuation between inspiration and expiration images. However, this correction was sufficient only when volume differences were much less than the difference between residual volume and FRC (20). Spirometrically gated air-trapping measures have been shown to be more sensitive than FEV1 in response to treatment in a cystic fibrosis cohort (21). Nevertheless, a recent study has shown that air trapping in heavy smokers with 3-month repeat exams have variability that is incompletely explained by breathing level (22). It has also been found that spirometry monitored with biofeedback aids in scanning children with cystic fibrosis at consistent lung levels for measuring air trapping (23). Although clinical CT protocols tend to undertake shorter scans to minimize x-ray exposure to the patient, simulations of reduced-dose scans by more widely spaced thin-slice images showed increased variability, but no more than typical of emphysema measured from such scans (24). We would expect PRM to show a similar responsiveness to low-dose CT scans or those reconstructed with iterative reconstruction methods, but this will require confirmation.

Longitudinal quantitative CT measures are also challenged by the normal fluctuations found in CT scanners as well as the lack of consensus on the appropriate reconstruction kernel. The trade-off is well known between CT image noise and resolution, and the variety of smoothing/denoising reconstruction kernels used in clinical practice has a confounding effect on histogram-quantified metrics such as PRM. Variations in calibration, reconstruction, and changes over time in scanners can shift reconstructed HU values, resulting in changes in the histograms of voxels within the lung VOI and therefore in the joint-density histogram of paired inspiration/expiration voxels as well. Such shifts lead to inconsistent classifications of voxels as normal, emphysematous, and air-trapping lung tissues. A previous study has shown that when torso HU values were tracked over time, tracheal air measures were variable, whereas air outside the abdomen was more stable (25). Gradual drifts in HUs showed up in phantom-based monitoring (26) with occasionally larger (>10 HU) shifts observed for both air and blood HU values (11). Adjustments for air and water values have been proposed based on actual measured HU values of blood (14) and/or air (27) but can be difficult because of the axial variability of air measures, particularly in the trachea. Tracheal air adjustments were useful for correcting some CT attenuation-based measures across scanners in a normal cohort, but possibly not for expiration-based measures (28). Different CT reconstruction kernels have been found to result in significant differences in mean LAA_−950I_ and LAA_−856E_ values (12) and have different noise levels (29, 30), and we found similar results with PRM metrics. Although comparing metrics from different reconstruction kernels has been attempted (31), it is not advisable for PRM. This variability caused by differing reconstruction methodology, particularly for quantifying small airways disease, remains a significant problem (11).

Because PRM depends on paired voxels from coregistered inspiration and expiration scans, the final source of variability in PRM is image registration. Advances in imaging software and registration methods have provided multiple robust methods for registering lung data sets, with accuracy typically at the subvoxel level (32, 33). When a lung registration succeeds, remaining misregistration instances are primarily local and small. Our simulations show that, possibly unexpectedly, such localized misregistrations introduce a smaller (< ±2%) variability than contributions from other scan acquisition factors. We observed that PRM robustness may be the most challenged when faced with more difficult registrations that are found in both healthier patients and those with a highly heterogeneous disease resulting from unusual deformations of the lung that are hard to recover. We have also confirmed that PRMs from different registration directions (ie, register inspiration to expiration vs register expiration to inspiration) have a small nonzero bias in PRM^fSAD^ values.

Some limitations to this study should be noted. The overall data presented for bias and noise characterization in the PRM CT methodology were acquired using a limited data set of 20 patients obtained from the COPDGene cohort. As such, future studies involving the use of larger longitudinal data sets (including zero-change/test-retest data) rather than the simulated data presented herein should be undertaken to demonstrate that corrective calibrations can be used to minimize nondisease-related variation. Furthermore, all potential sources of variation were investigated in isolation, whereas a multivariable analysis would be useful for evaluating their joint impact on PRM metrics.

As large COPD studies such as COPDGene and SPIROMICS begin to acquire longitudinal data, quantitative CT-based metrics must become robust enough to be able to analyze these data. To compare quantitative values, the sources of variability present in these measurements must be identified, and their effects on the quantitative measure must be further understood. Several factors that introduce variability into attenuation-based CT metrics can generally be avoided by standardizing CT protocols. Using the same CT parameters and reconstructions and registering these scans in a consistent direction each time lead to metrics with less variability (34). In summary, HU adjustment might not add sensitivity, standard reconstructions lead to less noisy PRM values, and registration direction should be consistent. Scans with fewer image slices do add noise, albeit small, to PRM metrics. However, PRM as a metric was shown to be most sensitive to lung volume, which may be influenced by changes in lung function as a result of disease progression as well as patient compliance. As such, care must be taken to ensure accurate ventilation during CT scanning because variability in lung volume remains difficult to correct.

## References

[1] Lynch DA, Al-Qaisi MA Quantitative computed tomography in chronic obstructive pulmonary disease. J Thoracic Imaging. 2013;28(5):284–290. 10.1097/RTI.0b013e318298733c PMC4161463 23748651

[2] Regan EA, Hokanson JE, Murphy JR, Make B, Lynch DA, Beaty TH, Curran-Everett D, Silverman EK, Crapo JD Genetic epidemiology of COPD (COPDGene) study design. COPD. 2010;7(1):32–43. 20214461 10.3109/15412550903499522 PMC2924193

[3] Couper D, LaVange LM, Han M, Barr RG, Bleecker E, Hoffman EA, Kanner R, Kleerup E, Martinez FJ, Woodruff PG, Rennard S Design of the subpopulations and intermediate outcomes in COPD Study (SPIROMICS). Thorax. 2014;69(5):491–494. 10.1136/thoraxjnl-2013-203897 PMC3954445 24029743

[4] Gevenois PA, de Maertelaer V, De Vuyst P, Zanen J, Yernault JC Comparison of computed density and macroscopic morphometry in pulmonary emphysema. Am J Respir Crit Care Med. 1995;152(2):653–657. 7633722 10.1164/ajrccm.152.2.7633722

[5] Gevenois PA, De Vuyst P, de Maertelaer V, Zanen J, Jacobovitz D, Cosio MG, Yernault JC Comparison of computed density and microscopic morphometry in pulmonary emphysema. Am J Respir Crit Care Med. 1996;154(1):187–192. 8680679 10.1164/ajrccm.154.1.8680679

[6] Matsuoka S, Kurihara Y, Yagihashi K, Hoshino M, Watanabe N, Nakajima Y Quantitative assessment of air trapping in chronic obstructive pulmonary disease using inspiratory and expiratory volumetric MDCT. Am J Roentgenol. 2008;190(3):762–769. 18287450 10.2214/AJR.07.2820

[7] Galbán CJ, Han MK, Boes JL, Chughtai KA, Meyer CR, Johnson TD, Galbán S, Rehemtulla A, Kazerooni EA, Martinez FJ, Ross BD Computed tomography-based biomarker provides unique signature for diagnosis of COPD phenotypes and disease progression. Nat Med. 2012;18(11):1711–1715. 23042237 10.1038/nm.2971 PMC3493851

[8] Müller NL, Staples CA, Miller RR, Abboud RT “Density mask.” An objective method to quantitate emphysema using computed tomography. Chest. 1988;94(4):782–787. 3168574 10.1378/chest.94.4.782

[9] Pilgram TK, Quirk JD, Bierhals AJ, Yusen RD, Lefrak SS, Cooper JD, Gierada DS Accuracy of emphysema quantification performed with reduced numbers of CT sections. Am J Roentgenol. 2010;194(3):585–591. 20173132 10.2214/AJR.09.2709 PMC2838241

[10] Galbán CJ, Boes JL, Bule M, Kitko CL, Couriel DR, Johnson TD, Lama V, Telenga ED, van den Berge M, Rehemtulla A, Kazerooni EA, Ponkowski MJ, Ross BD, Yanik GA Parametric response mapping as an indicator of bronchiolitis obliterans syndrome following hematopoietic stem cell transplantation. Biol Blood Marrow Transplant. 2014;20(10):1592–1598. 24954547 10.1016/j.bbmt.2014.06.014 PMC4163140

[11] Sieren JP, Newell JD, Judy PF, Lynch DA, Chan KS, Guo J, Hoffman EA Reference standard and statistical model for intersite and temporal comparisons of CT attenuation in a multicenter quantitative lung study. Med Phys. 2012;39(9):5757–5767. 22957640 10.1118/1.4747342 PMC3448623

[12] Wilson C, Williams A, Stinson D, Estepar R, Washko G, Sieren J, Tschirren J, Hoffman EA, Lynch DA Impact of differing convolution kernels on quantitative CT measures of lung density and correlation with physiology in smokers: B31f Vs B35f. Am J Respir Crit Care Med. 2012;185:A2029.

[13] Rabe KF, Hurd S, Anzueto A, Barnes PJ, Buist SA, Calverley P, Fukuchi Y, Jenkins C, Rodriguez-Roisin R, van Weel C, Zielinski J Global strategy for the diagnosis, management, and prevention of chronic obstructive pulmonary disease: GOLD executive summary. Am J Respir Crit Care Med. 2007;176(6):532–555. 17507545 10.1164/rccm.200703-456SO

[14] Stoel BC, Vrooman HA, Stolk J, Reiber JH Sources of error in lung densitometry with CT. Invest Radiol. 1999;34(4):303. 10196723 10.1097/00004424-199904000-00008

[15] Kim SS, Seo JB, Kim N, Chae EJ, Lee YK, Oh YM, Lee SD Improved correlation between CT emphysema quantification and pulmonary function test by density correction of volumetric CT data based on air and aortic density. Eur J Radiol. 2014;83(1):57–63. 22613510 10.1016/j.ejrad.2012.02.021

[16] Gorbunova V, Sporring J, Lo P, Loeve M, Tiddens HA, Nielsen M, Dirksen A, de Bruijne M Mass preserving image registration for lung CT. Med Image Anal. 2012;16(4):786–795. 22336692 10.1016/j.media.2011.11.001

[17] Madani A, Van Muylem A, Gevenois PA Pulmonary emphysema: effect of lung volume on objective quantification at thin-section CT 1. Radiology. 2010;257(1):260–268. 20663967 10.1148/radiol.10091446

[18] Keller BM, Reeves AP, Henschke CI, Yankelevitz DF Multivariate compensation of quantitative pulmonary emphysema metric variation from low-dose, whole-lung CT scans. Am J Roentgenol. 2011;197(3):W495–W502. 21862778 10.2214/AJR.11.6444

[19] Park SJ, Lee CH, Goo JM, Heo CY, Kim JH Inter-scan repeatability of CT-based lung densitometry in the surveillance of emphysema in a lung cancer screening setting. Eur J Radiol. 2012;81(4):e554–e560. 21752566 10.1016/j.ejrad.2011.06.028

[20] Goris ML, Zhu HJ, Blankenberg F, Chan F, Robinson TE An automated approach to quantitative air trapping measurements in mild cystic fibrosis. Chest. 2003;123(5):1655–1663. 12740287 10.1378/chest.123.5.1655

[21] Robinson TE, Goris ML, Zhu HJ, Chen X, Bhise P, Sheikh F, Moss RB Dornase alfa reduces air trapping in children with mild cystic fibrosis lung disease: a quantitative analysis. Chest. 2005;128(4):2327–2335. 16236891 10.1378/chest.128.4.2327

[22] Mets OM, de Jong PA, van Ginneken B, Gietema HA, Lammers JW Quantitative computed tomography in COPD: possibilities and limitations. Lung. 2012;190(2):133–145. 22179694 10.1007/s00408-011-9353-9 PMC3310986

[23] Kongstad T, Buchvald FF, Green K, Lindblad A, Robinson TE, Nielsen KG Improved air trapping evaluation in chest computed tomography in children with cystic fibrosis using real-time spirometric monitoring and biofeedback. J Cyst Fibros. 2013;12(6):559–566. 23810566 10.1016/j.jcf.2013.05.012

[24] Dirksen A, Friis M, Olesen K, Skovgaard L, Sorensen K Progress of emphysema in severe α1-antitrypsin deficiency as assessed by annual CT. Acta Radiol. 1997;38(5):826–832. 9332238 10.1080/02841859709172418

[25] Judy PF, Nawfel RD, Silverman SG Systematic scanner variability of patient CT attenuation measurements. SPIE Proceedings. Vol. 7258: Medical Imaging: International Society for Optics and Photonics; 2009.

[26] Stoel BC, Bode F, Rames A, Soliman S, Reiber JH, Stolk J Quality control in longitudinal studies with computed tomographic densitometry of the lungs. Proc Am Thorac Soc. 2008;5(9):929–933. 19056719 10.1513/pats.200804-039QC

[27] Parr DG, Stoel BC, Stolk J, Nightingale PG, Stockley RA Influence of calibration on densitometric studies of emphysema progression using computed tomography. Am J Respir Crit Care Med. 2004;170(8):883–890. 15271692 10.1164/rccm.200403-326OC

[28] Zach JA, Newell JD Jr , Schroeder J, Murphy JR, Curran-Everett D, Hoffman EA, Westgate PM, Han MK, Silverman EK, Crapo JD, Lynch DA Quantitative computed tomography of the lungs and airways in healthy nonsmoking adults. Invest Radiol. 2012;47(10):596–602. 22836310 10.1097/RLI.0b013e318262292e PMC3703944

[29] Schilham A, van Ginneken B, Gietema H, Prokop M Local noise weighted filtering for emphysema scoring of low-dose CT images. IEEE Trans Med Imaging. 2006;25(4):451–463. 16608060 10.1109/TMI.2006.871545

[30] Hochhegger B, Irion KL, Marchiori E, Moreira JS Reconstruction algorithms influence the follow-up variability in the longitudinal CT emphysema index measurements. Korean J Radiol. 2011;12(2):169–175. 21430933 10.3348/kjr.2011.12.2.169 PMC3052607

[31] Bartel ST, Bierhals AJ, Pilgram TK, Hong C, Schechtman KB, Conradi SH, Gierada DS Equating quantitative emphysema measurements on different CT image reconstructions. Med Phys. 2011;38(8):4894–4902. 21928661 10.1118/1.3615624 PMC3165435

[32] Murphy K, van Ginneken B, Reinhardt J, Kabus S, Ding K, Deng X, Cao K, Du K, Christensen GE, Garcia V, Vercauteren T, Ayache N, Commowick O, Malandain G, Glocker B, Paragios N, Navab N, Gorbunova V, Sporring J, de Bruijne M, Han X, Heinrich MP, Schnabel JA, Jenkinson M, Lorenz C, Modat M, McClelland JR, Ourselin S, Muenzing SE, Viergever MA, De Nigris D, Collins DL, Arbel T, Peroni M, Li R, Sharp GC, Schmidt-Richberg A, Ehrhardt J, Werner R, Smeets D, Loeckx D, Song G, Tustison N, Avants B, Gee JC, Staring M, Klein S, Stoel BC, Urschler M, Werlberger M, Vandemeulebroucke J, Rit S, Sarrut D, Pluim JP Evaluation of methods for pulmonary image registration: the EMPIRE10 study. IEEE Trans Med Imaging. 2011;30(11):1901–1920. 21632295 10.1109/TMI.2011.2158349

[33] Castillo R, Castillo E, Fuentes D, Ahmad M, Wood AM, Ludwig MS, Guerrero T A reference data set for deformable image registration spatial accuracy evaluation using the COPDgene study archive. Phys Med Biol. 2013;58(9):2861–2877. 23571679 10.1088/0031-9155/58/9/2861 PMC3677192

[34] Newell JD Jr., Sieren J, Hoffman EA Development of quantitative computed tomography lung protocols. J Thorac Imaging. 2013;28(5):266–271. 23934142 10.1097/RTI.0b013e31829f6796 PMC3876949

